# Migration and health: fact, fiction, art, politics

**DOI:** 10.1186/1742-7622-3-15

**Published:** 2006-10-02

**Authors:** Clarence C Tam

**Affiliations:** 1 Infectious Disease Epidemiology Unit, Department of Epidemiology and Population Health, London School of Hygiene & Tropical Medicine, London, UK; 2 Environmental and Enteric Diseases Department, Health Protection Agency Centre for Infections, London, UK

## Abstract

The recent Immigration Bill debate in the United States Congress has again re-ignited the polemic regarding immigration policy. In this essay, I argue that disputes surrounding the legality of migrant workers highlight chronic, underlying problems related to factors that drive migration. The public health field, although concerned primarily with addressing the health needs of migrant populations, cannot remain disengaged from the wider debates about migration. The health needs of migrants, although in themselves important, are merely symptoms of deeper structural process that are intrinsically linked to equity and human rights, and simply focusing on health issues will be insufficient to address these societal pathologies.

"Therefore is the name of it called Babel; because the Lord did there confound the language of all the earth: and from thence did the Lord scatter them abroad upon the face of all the earth."

Genesis 11: 9.

The Whitney Museum of American Art in New York recently held its Biennial 2006 exhibition [1]. Entitled *Day for Night*, after the Truffaut film in which scenes shot in daylight are made to imply night-time, the exhibition was guided by themes of ambiguity, in the literal (works that are not quite what they appear to be), conceptual (in deconstructing the role of the artist in the current socio-political climate) and practical senses (in trying to define what actually constitutes "American" art). The 2006 Biennial was heavily influenced by the work of appropriationists, artists reclaiming everyday objects to create artistic statements. In that vein, I have "appropriated" three objects of my own in this discussion about migration: one artistic (Mark Bradford's *Los Moscos*), one literary (Upton Sinclair's *The Jungle*), and one political (the proposed Immigration Bills currently under consideration in the United States Congress). These three objects make different comments on migration – specifically worker migration – and provide "extra-scientific" perspectives that reveal certain causal truths not immediately apparent from a purely scientific approach. This essay focuses on the intersection between migration and health not to comment on the health of migrant workers, but rather to argue that the issue of migrant health is merely a symptom of a much wider socio-political discussion, from which the public health community is noticeably absent, but in which it must engage if the rights of those most affected by current social and political trends are to be protected. I first describe each of my three appropriations in turn and subsequently comment on the implications for the public health community. Although I focus on the United States (US) context – the source of my three appropriations – my discussion is general and relevant to other settings.

Los Angeles artist Mark Bradford's *Los Moscos *(*The Flies*, see Figure 1) [2] takes its name from a derogatory term referring to California's illegal labourers. At first glance, this collage suggests a view of the city's night lights from the air. On closer inspection, however, the work reveals far deeper ironies: the radial patterns visible throughout the canvas (for example, Figure 1) suggest helicopter blades over a city constantly under surveillance, yet the very individuals whom the work is intended to portray remain unseen. This metaphorical frustration – an existent yet invisible population – is expressed quite literally; the collage is built from segments of billboard advertisements and "street spam", which seem somehow familiar but difficult to place. Even more ironically, the most familiar yet unrecognizable parts are the black portions of the canvas, made up from segments of Apple's billboards for its iPod products.

Such ironies are the frank reality for the public health community – the most needy populations are usually the least accessible, whether for legal, political, social, cultural or programmatic reasons. It is precisely in such an environment – the need to comment and act upon the health of certain populations given scant evidence – that we most risk making inaccurate statements and implementing misguided policies. Mark Bradford's work warns us of the pitfalls of vivid colours and bright lights, drawing us instead towards the darkness and inviting us to focus more closely on those "living under the radar of formal business" [3].

If Mark Bradford's *Los Moscos *portrays the reality of those looking into the darkness, then Upton Sinclair's *The Jungle *[4] describes the reality for those living in it. In his incisive critique of the meat industry and the deplorable working conditions of its workers, Sinclair follows the plight of an aspiring family of Lithuanian immigrants who seek employment in the thriving, but ultimately destructive, stockyards of Chicago. The book was instrumental in exposing the unsanitary practices of the meat industry, culminating in the Roosevelt-endorsed Federal Meat Inspection Act of 1906. For all the ensuing uproar regarding the safety of meat intended for human consumption, however, Sinclair's intended purpose – to highlight the exploitation of workers – received little attention, a fact he himself recognized in the now-famous quotation: *"I aimed at the public's heart, and by accident I hit it in the stomach." *Indeed, most of the book is concerned with cataloguing, in unsparingly clinical detail, the succession of financial, physical, psychological insults suffered by its protagonists: the discrimination, the unfair wages, the dire working conditions, the debt slavery, the poor housing, the physical abuse, the unhygienic food, the poisonous medicines. This sequence of events is embodied in a series of tragic and, to the reader, seemingly inevitable outcomes – repeated injuries, chronic poisonings, arrest, death – the conditions for which were undoubtedly already present even before the story began. Sinclair's account, however, is neither merely documentary nor impassive; the victim of this series of misfortunes is also the victimized:

*"But no, their bells were not ringing for him – their Christmas was not meant for him, they were simply not counting him at all. He was of no consequence; he was flung aside, like a bit of trash, the carcass of some animal. It was horrible, horrible! His wife might be dying, his baby might be starving, his whole family might be perishing in the cold – and all the while they were ringing their Christmas chimes! And the bitter mockery of it – all this was punishment for him! They put him in a place where the snow could not beat in, where the cold could not eat through his bones; they brought him food and drink – why, in the name of Heaven, if they must punish him, did they not put his family in gaol and leave him outside?" *[[4], p. 192]

And while the protagonist might bemoan and wonder at his misfortunes, the author is in no doubt as to their causal structure:

*"He had no wit to trace back the social crime to its far sources – he could not say that it was the thing men have called 'the system' that was crushing him to the earth; that it was the packers, his masters, who had bought up the law of the land, and had dealt out their brutal will to him from the seat of justice." *[[4], p. 193]

Sinclair's narrative is thus ultimately not a comment on ill-health, his focus is not on injuries and deaths, but is rather a thesis on the structural violence that gives rise to them among unorganized individuals within highly organized systems.

It is in this context, compounded by national security concerns, that the United States Congress currently finds itself at an impasse over proposed reforms to the country's immigration laws. In its original form, passed by the House of Representatives last December, the bill included provisions for erecting several hundred miles of fencing along the US-Mexico border and classifying illegal immigrants as felons. These two factors proved to be major points of contention in the Senate debate, as did the possibility of amendments incorporating a so-called "guest-worker" programme [5,6]. Intense opposition to the criminalization of immigrants led to large-scale demonstrations among the Hispanic communities most likely to be affected, and to calls for legalizing the considerable number of illegal immigrants already in the country [7]. With the prospect of legislative changes that could offer routes to legal employment and eventual citizenship, the Mexican border has already begun to see a rush of hopefuls planning to cross into the US in time to qualify for any guest-worker programme and before the tightening of border controls [8].

That the US should find itself in such an unenviable position is unsurprising. For decades, successive administrations have implemented inconsistent and haphazardly-enforced immigration policies that have nevertheless parallelled a continual increase in the influx of migrant labour [9,10]. Periodic threats of a tightening of border controls have merely encouraged the permanence of net immigration, as workers who might otherwise return to their home countries decide to stay for fear of being permanently separated from family already settled in the US [11]. Stricter border controls, more extensive background checks and criminalization of unauthorized entry will have little effect on immigration without addressing more fundamental issues driving such movement; amendments such as guest-worker programmes are merely stop-gaps indicative of deeper societal pathologies.

The heavy reliance on temporary foreign labour, primarily in food production, building and the service industry, has resulted in a chronic dependency on immigrants workers, many of them unauthorized, to support these sectors. Over 70% of farm crop workers in the US are foreign-born, primarily from Mexico; more than 50% of these are unauthorized to work in the US [12]. The average farm worker is 33 years old, has a sixth grade education, speaks and reads little or no English and earns $10 an hour undertaking physically demanding and, in many cases, hazardous work for 34 weeks of the year, with little recourse to other employment, either in the off-season or in the long term [12,13]. Thirty percent of farmworker households earn below the poverty line, and over 75% of workers have no medical cover. Despite this, approximately one quarter have worked in the US under their current employer for at least five years [12]. The commonly-heard assertion that foreign labour fills jobs that Americans are unwilling to do is simply untrue; these are jobs that Americans simply cannot afford to take.

Although current regulations require employers to verify the legality of their employees, the current structure of the food industry provides little incentive to do so. For every dollar spent on food in the US, an average of 20 cents returns to the grower; for fruit and vegetable farmers, the gross return can be as little as five cents to the dollar [14]. The issue is compounded by outdated and misdirected federal policies on agricultural subsidies to promote the growth of crops such as wheat, soybeans and corn, whose production cost is minimal relative to the cost of processing their derivatives, including sweeteners and hydrogenated fats used in processed and "value-added" foods [16]. By contrast, perishable fruits and vegetables are highly sensitive to price variations, yet receive limited federal support. Farmers thus have limited power to attract local labour by increasing wages, and many of these commodities have limited potential for efficiency gains through mechanization, leading instead to what has been referred to as the "Mexicanization" of agriculture [13]. Farm work, although one of the lowest-paid jobs in the US, still pays an average daily wage over 15 times that of equivalent jobs south of the border [16].

The low returns from agriculture encourage over-production of food, keeping prices artificially low for consumers and encouraging increased consumption. The US produces approximately 3500 calories per person per day, a quarter of which is lost to spoilage and waste [14,17]. The average American consumes over 2500 calories per day, yet spends 10% of their disposible income on food, less than in any other country [14,18]. The myth of a seemingly limitless supply of cheap food means that consumers are purchasing, and expecting, food at highly subsidized and unrealistic prices. Manufacturers and retailers, by virtue of their market share and product flexibility, have the power to shift some of these costs to producers and these, in turn, offset costs by transferring them onto workers who, being at the bottom of a long chain of false economies, have no one left to subsidize them. Meanwhile, euphemisms such as "guest-worker" programmes are merely veiled admissions of a society's unwillingness to bite the hand that quite literally feeds its gluttony, instead maintaining an underclass of individuals "living under the radar of formal business" who bolster its bloated economy, but whose rights and contributions to society are largely ignored.

So how does this impact on the public health community? An oft-heard assertion has it that population movement is a natural consequence of current "globalizing" trends [19,20]. However, uncritical acceptance of such apparently benign statements merely serves to support some particularly pernicious realities. There is nothing natural about many, if not most, types of population movement taking place today. I do not intend to provide an immediate solution to the current structure of the food supply here; I wish instead to argue that even a brief analysis reveals that the health issues of migrant farm workers are inextricably linked to the politics, economics and culture of food. Such analyses can and should be done not only in the context of worker migration, but also for other forms of migration [21] and, indeed, other health contexts [22] . Invariably, these point to the insufficiencies of focusing exclusively on ill-health [22]. Public health should not merely constitute the maintenance of a state of "absence of disease", but should be a pro-active enterprise striving for equity and social justice, with human rights at its core and "health" as the main intended outcome of such activity. By limiting our remit to dealing with ill-health, we risk making our field subservient to the often contradictory interests of politics and economics and, in many cases, even financing their excesses.

The recent success of global industry has relied largely on the development of "liberalizing" trade policies, enabling private enterprises to transcend the nation state and seek resources and labour to suit their economies. In today's world, an American product is American only in the ideal. As the Whitney Biennial demonstrates, American art need not be conceived by an American artist, created in the US from American materials, or even be thematically "American" – the idea of American art transcends all borders. Despite recent specific and well-directed global health initiatives, however, the public health field has lagged behind in this respect, remaining to date primarily a statist enterprise largely secondary to the interests and politics of the state. As Farmer forcefully argues, *"those who direct modern commerce are far ahead of us. They understand the artificiality of borders and the gains to be made from differentials in price and supply; they exploit the whole world. Meanwhile, the forces of healing, which deal in the priceless and universal value of health, are trammeled by parochialisms of place and creed." *[23]

If current trends persist, our adherence and high dependence on the nation state will make it increasingly difficult for us to meet the health needs of mobile populations, particularly those whose health interests are not recognized by any nation. For public health to be a successful endeavour, it too must be an idea that transcends borders, a global enterprise guided not by the interests of states and corporations, but by the needs of those most disenfranchised in society. The health of migrant populations is important not because migrants represent groups with different health outcomes, not because they are difficult populations to access, not because special methodologies are required to study their health needs or deliver interventions, but because they, like everyone else, have basic entitlements codified under the Universal Declaration of Human Rights [24]. Viewed from this perspective, adverse outcomes in migrant populations become the physical and psychological embodiments of a long and complex combination of structural factors so acutely captured by Upton Sinclair a century ago; disease profiles and methodological issues are mere details, albeit very important details, in such a system. To once again quote Farmer:

*"To argue that human rights abuses [...] are unrelated to our surfeit in the rich world requires that we erase history and turn a blind eye to the pathologies of power that transcend all borders. Perpetuating such fictions requires dishonest, desocialized analyses that mask – whether through naïveté or fecklessness or complicity – the origins and consequences of structural violence [...] it is time to take health rights as seriously as other human rights, and that intellectual recognition is only a necessary first step toward pragmatic solidarity, that is, toward taking a stand by the side of those who suffer most from an increasingly harsh "new world order." *[23]

**Figure 1:**
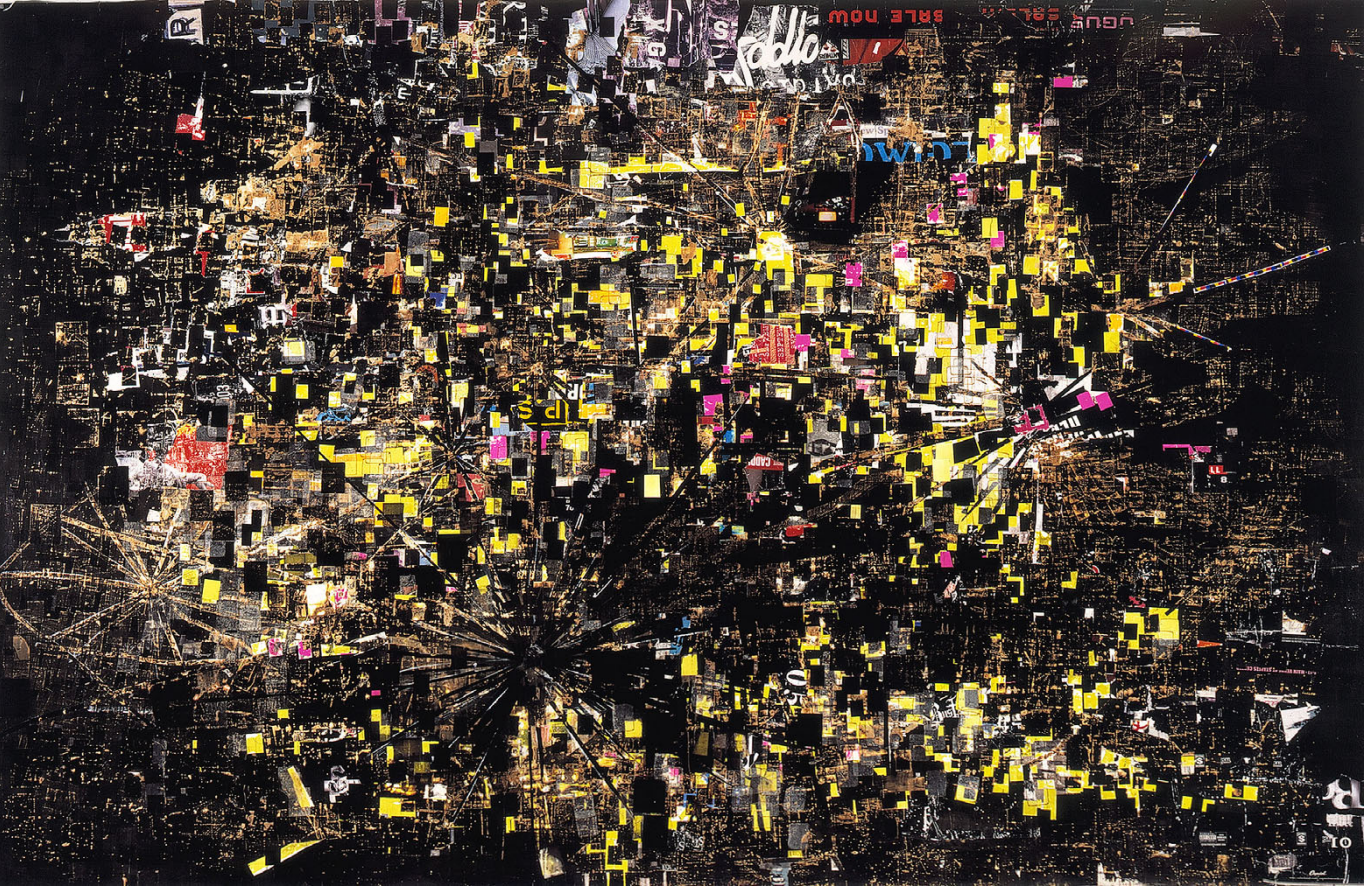
Mark Bradford, *Los Moscos *2004, Mixed media on canvas, 125 × 190 1/2 inches. Sikkema Jenkins & Co.
